# Association between head injury and concussion with retinal vessel caliber

**DOI:** 10.1371/journal.pone.0200441

**Published:** 2018-07-11

**Authors:** Bamini Gopinath, Gerald Liew, Ashley Craig, Ilaria Pozzato, Susanne Meares, George Burlutsky, Ian D. Cameron, Paul Mitchell

**Affiliations:** 1 Centre for Vision Research, Department of Ophthalmology and Westmead Institute for Medical Research, University of Sydney, Sydney, NSW, Australia; 2 John Walsh Centre for Rehabilitation Research, Sydney Medical School, Kolling Medical Research Institute, University of Sydney, Sydney, NSW, Australia; 3 Department of Psychology, Macquarie University, Sydney, NSW, Australia; University of Melbourne, AUSTRALIA

## Abstract

The adverse long-term consequences following traumatic brain injury are poorly understood, particularly on the cerebral microvasculature. Retinal vessels are a surrogate marker of cerebral vascular changes. We therefore aimed to examine the cross-sectional association between serious head injury or being knocked unconscious, and/or concussion and retinal microvascular signs, specifically, mean retinal arteriolar and venular calibre, in older adults after accounting for potential confounders. This cohort study involved 2,624 adults with mean age of 66.9 (±9.1) years who self-reported head injury and concussion parameters, and had gradable retinal photographs. Face-to-face interviews with trained interviewers allowed participants to report prior serious head injury or being knocked unconscious, and/or a previous diagnosis of concussion by a medical professional. Fundus photographs were taken and retinal vascular calibre measured using computer-assisted techniques and summarized. There were 25.9%, 15.3% and 10.1% who reported a prior serious head injury or being “knocked unconscious”, concussion, and both, respectively. Participants in the first group compared to non-injured participants had significantly wider (~2 μm) mean retinal venular calibre (p = 0.02), after adjusting for age, sex, smoking, body mass index, mean arterial blood pressure, type 2 diabetes and fellow vessel calibre. No significant associations were observed in people reporting medically diagnosed concussion or with mean retinal arteriolar calibre. Our exploratory study suggests that head injury is independently associated with wider retinal venular caliber. These findings warrant further investigation in longitudinal cohort studies.

## Introduction

Traumatic brain injury (TBI) is a significant cause of hospital admission for older adults [1]. Falls are the most frequent cause of hospital admission for older adults, and also the most common mechanism for TBI [1, 2]. A range of problems can persist after TBI, including post-concussion symptoms, emotional difficulties, cognitive impairment, and functional limitations [3, 4]. The long-term consequences of TBI are a matter of substantial concern for affected individuals and their families, and for society due to the substantial economic burden [4, 5]. Further, the costs are greater in older adults and set to increase with the aging demographics of most countries [4, 5]. Mortality resulting from TBI also increases with age, rising from 71% in 65–70-year-old patients to 87% for patients >80 years [6]. Therefore, objective imaging and prognostic indicators of neurodegeneration and other poor health outcomes related to TBI or head injury in older adults are needed, as evidence suggests that such biomarkers are imperative for accurate diagnosis and for targeting therapy [4, 7].

In this context, the link between retinal pathology and a variety of cerebrovascular and neurodegenerative diseases has been established by multiple studies [8]. Several clinic- and population-based studies show that cerebrovascular pathology has modest correlations with structural changes in the retinal microvasculature, supporting the hypothesis that the retina may be a ‘window to the brain’[9]. By performing retinal photography, subtle retinal microvascular changes have been established in stroke, dementia, cognitive dysfunction, depression and anxiety [10–14]. To our best knowledge, only one study has examined the associations between TBI and retinal microvascular signs. A recent Singaporean study conducted within an emergency department, found that mild TBI patients compared to controls had increased retinal arterial and venous tortuosity (indicating increased curvature of retinal vessels) persisting for a period of 6 months after the injury [15]. Further investigation and understanding of the pathological alterations of retinal microvessels may be valuable for understanding the adverse long-term consequences following TBI. Therefore, the current study used a relatively large cohort of adults aged 50+ years to examine the cross-sectional association between self-reported measures of serious head injury/ being knocked unconscious and/or concussion, with alterations to the retinal microvascular structure; specifically, changes to mean retinal arteriolar and venular calibre.

## Methods

### Study population

The Blue Mountains Hearing Study is a population-based survey of age-related hearing loss conducted during the years 1997–2004 among participants of the Blue Mountains Eye Study (BMES) cohort [16]. During 1992–4, 3654 participants aged 49+ years were examined (82.4% participation; BMES-1). Surviving baseline participants were invited to attend 5-year follow-up examinations (1997–9, BMES-2), at which 2334 (75.1% of survivors) and an additional 1174 newly eligible residents were examined. Hearing was measured at BMES-2 i.e. 2956 participants aged 50+ years had audiometric testing performed. The current cross-sectional analyses involved participants from BMES-2 only (n = 2956) as questions around head injury/ being knocked unconscious and concussion were administered to only those who had had their hearing function tested. The study was approved by the Human Research Ethics Committee of the University of Sydney and was conducted adhering to the tenets of the Helsinki Declaration. Signed informed consent was obtained from all the participants at each examination.

### Assessment of head injury and concussion

At BMES-2 as part of the hearing questionnaire, participants were asked to respond ‘yes’ or ‘no’ to the following questions: 1) ‘Have you had any serious head injuries or have you been knocked unconscious?” and 2) ‘Has a doctor ever told you that you had concussion?’ Therefore, cross-sectional analysis involved three groups of participants i.e. those classified as having had: 1) sustained a serious head injury or been knocked unconscious; 2) doctor-diagnosed concussion; and 3) serious head injury/ knocked unconscious as well as doctor diagnosed concussion (answered ‘yes’ to both the above questions).

### Retinal photography

Methods for grading the caliber of retinal arterioles and venules are described elsewhere [17]. In brief, at the baseline examination, 30° photographs of the macula, optic disc, and other retinal fields of both eyes were taken, after pupil dilation, using a Zeiss FF3 fundus camera (Zeiss, Oberkochen, Germany). We used methods developed by the University of Wisconsin–Madison [17], to measure the internal caliber of retinal arterioles and venules from digitized photographs. These were then summarized using established formulas [18] that account for branching patterns and combine individual vessel calibers into summary indices, and are presented as the central retinal artery equivalent (CRAE) or central retinal vein equivalent (CRVE), representing the mean caliber of these vessels. Intra- and inter-grader reliability of this method was high [18], with quadratic weighted κ values of 0.85 (CRAE) and 0.90 (CRVE) found for inter-grader reliability and between 0.80 to 0.93 and 0.80 to 0.92 for intra-grader reliability of the two graders, respectively. Vessel diameters for right eyes only were used in the analyses.

### Assessment of potential confounders

At face-to-face interviews with trained interviewers, a comprehensive medical history that included information about demographic factors, socio-economic characteristics and lifestyle factors like smoking, was obtained from all participants. History of smoking was defined as never, past, or current smoking. Current smokers included those who had stopped smoking within the past year. Body mass index (BMI) was calculated as weight divided by height squared (kg/m^2^). Blood pressure (BP) was measured using standard auscultatory methods. Mean arterial BP (mm Hg) was defined as 0.33×systolic BP + 0.67×diastolic BP. Diabetes was defined either by history or from fasting blood glucose ≥7.0 mmol/L. These potential confounders were adjusted for in multivariable analysis as these have been shown to influence independently retinal vessel calibre in the BMES [16, 19].

### Statistical analysis

SAS statistical software (SAS Institute, Cary NC) version 9.4 was used for linear regression analyses. Linear regression models were constructed to examine cross-sectional associations between serious head injury/ knocked unconscious and/ or concussion (independent variables) with retinal vascular caliber (dependent variable). Serious head injury/ knocked unconscious and/or concussion were analyzed as dichotomized variables (yes/ no). Analysis of covariance was used to calculate differences in mean retinal vascular caliber adjusted for age, sex, smoking, BMI, mean arterial BP, diabetes and fellow vessel caliber. To assess the retinal vessel caliber values while avoiding collinearity between arteriolar and venular diameter [20], we adjusted for fellow vessel calibre in the final model i.e. adjusted arteriolar diameter for venular diameter and venular diameter for arteriolar diameter using the residual method previously described by Willett [21].

## Results

Of the 2956 participants examined at BMES-2, 298 participants did not have retinal vessel data collected; 30 participants did not answer the questions on serious head injury/ concussion; and 4 participants did not have both retinal vessel calibre or serious head injury/ concussion data. Hence, a total of 332 participants were excluded, leaving 2624 participants with complete data who were included in the final cross-sectional analysis. Table 1 shows the study characteristics of these participants. Around one in four participants reported experiencing a serious head injury or being knocked unconscious (25.9%), and 15.3% reported that a doctor had diagnosed them with a concussion. Around one in ten participants reported having a serious head injury/ being knocked unconscious as well as doctor-diagnosed concussion (Table 1).

**Table 1:** Study characteristics of participants (n = 2624).

Characteristics	Participants
Age, *yrs*	66.9 (9.1)
Males	1123 (42.8)
Current smokers	252 (9.7)
Body mass index, *kg/m*^*2*^	27.7 (4.8)
Mean arterial blood pressure, *mmHg*	105.2 (12.4)
History of diabetes	262 (10.0)
Mini Mental State Exam scores	28.6 (2.0)
Serious head injury or knocked unconscious	680 (25.9)
Doctor diagnosed concussion	400 (15.3)
Head injury and concussion	266 (10.2)
Mean retinal arteriolar caliber, *μm*	188.3 (14.6)
Mean retinal venular caliber, *μm*	224.9 (16.4)

Data are presented mean (SD) or as n (%) unless otherwise stated.

Table 2 shows that after multivariable adjustment, participants who were reported sustaining a serious head injury or being knocked unconscious compared to those who were not injured had significantly wider retinal venular caliber (~2 μm; p = 0.02). No other significant associations were observed with retinal arteriolar caliber or in those who reported doctor-diagnosed concussion (Table 2).

**Table 2:** Cross-sectional association between head injury, concussion and retinal vessel caliber in older adults (n = 2624).

	Retinal arteriolar calibre, mean (SE)		Retinal venular calibre, mean (SE)	
Head injury type	Age-sex-adjusted	Multivariable-adjusted ^a^	Age-sex-adjusted	Multivariable-adjusted ^a^
Serious head injury or knocked unconscious				
No (n = 1944)	188.5 (0.33)	188.5 (0.34)	224.4 (0.37)	224.5 (0.38)
Yes (n = 680)	187.8 (0.57)	187.5 (0.57)	226.5 (0.53)	226.3 (0.64)
p-value	0.34	0.13	**0.003**	**0.02**
Concussion				
No (n = 2216)	188.3 (0.31)	188.2 (0.31)	224.9 (0.35)	225.0 (0.35)
Yes (n = 400)	188.3 (0.73)	188.2 (0.74)	225.2 (0.82)	225.0 (0.83)
p-value	0.98	1.00	0.72	0.98
Severe head injury/ knocked unconscious and concussion				
No (n = 2350)	188.3 (0.30)	188.3 (0.31)	224.7 (0.34)	224.8 (0.34)
Yes (n = 266)	188.2 (0.90)	188.1 (0.78)	226.5 (1.00)	226.1 (1.01)
p-value	0.94	0.85	0.09	0.22

^a^ Further adjusted for body mass index, smoking, mean arterial blood pressure, type 2 diabetes, and fellow vessel caliber.

Supplementary analysis involved excluding participants who had cognitive impairment, as individuals with impaired cognition are subsequently more likely to experience falls and/or TBI. Cognitive function was assessed in the BMES using the mini-mental state exam (MMSE), with MMSE scores <24 indicating cognitive impairment [22]. There were 75 participants with cognitive impairment who were excluded from analysis. This exclusion did not markedly change the observed estimates, that is, participants reporting a serious head injury or being knocked unconscious compared to the non-injured group had significantly wider retinal venular calibre, 226.3 and 224.6 μm, respectively (multivariable-adjusted p = 0.02). All other associations remained non-significant.

## Discussion

This study provides novel epidemiological data suggesting that older adults with prior serious head injury or experience of being knocked unconscious had significantly wider retinal venular caliber, which is an adverse retinal microvascular sign. This association persisted after adjusting for potential confounders such as age, sex, smoking, body mass index, blood pressure and type 2 diabetes. Exclusion of participants with cognitive impairment also did not markedly change this observed association with retinal venular caliber. However, serious head injury/ being knocked unconscious and/or concussion were not independently associated with mean retinal arteriolar caliber.

The prevalence of serious head injury and/or being knocked unconscious or reporting only a concussion ranged from 25.9% to 10.1% in BMHS participants aged 50+ years and this broadly concurs with other studies from China [23] and Australia [24] which reported prevalence rates of around 19% and 24.1% of TBI and concussion, respectively, but our rates are lower than the 45% reported in a UK cohort of older trauma patients [25].

Our finding of an independent association between head injury and wider retinal venular caliber contrasts with the non-significant findings related to retinal venules observed in mild TBI patients from the Singaporean study [15]. However, our observation is supported by a systematic review and individual-participant meta-analysis, which reported that venules might play a significant role in the pathogenesis of stroke (a major cause of falls and subsequent TBI) after confirming that wider retinal venular diameter, rather than narrower arteriolar caliber, is more strongly related to stroke risk [9, 26–28]. Hence, it is possible that the link between serious head injury or being knocked unconscious and retinal venules could be mediated by stroke or other ischemic conditions elsewhere in the body, such coronary heart disease or limb ischemia [29, 30]. We also previously showed that cerebral hemorrhages were associated with significant widening of retinal venules [31]. Additionally, only wider retinal venules were observed in individuals who developed schizophrenia, suggesting this represents microvascular abnormality reflective of deficient brain oxygenation [32]. More recently, enlarged perivascular spaces (ePVSs) in the brain, have emerged as a promising imaging biomarker for vascular brain pathology [20], and wider retinal venular caliber was associated with more ePVSs, independently of structural brain magnetic resonance imaging (MRI) markers and vascular risk factors [28]. Commonalities between the above neurovascular/ neurodegenerative diseases and TBI and/or overlapping vascular brain pathology, lends plausibility to our observation of wider retinal venules among participants reporting a serious head injury or being knocked unconscious.

The specific pathophysiological mechanisms underlying wider retinal venular caliber are not fully understood [32, 33]. It has been hypothesized that wider venules reflect cumulative structural damage to the microvasculature (for example, from inflammation or endothelial dysfunction) and wider retinal venular caliber could be a result of hypoxia/ ischemia [32–34]. TBI induces a complex array of immunological/ inflammatory tissue responses similar to ischaemic reperfusion injury [35]. Moreover, TBI is characterized by an imbalance between cerebral oxygen delivery and cerebral oxygen consumption. Although this mismatch is induced by various different vascular and haemodynamic mechanisms, the final common endpoint is brain tissue hypoxia [35]. These pathophysiological events associated with TBI could also explain our observed associations with wider retinal venules among older adults reporting a serious head injury/ being knocked unconscious. Alternatively, genetic factors influence retinal venular caliber [36, 37]. Genetic linkage regions for venular caliber are implicated in endothelial function and vasculogenesis [36, 37]. Thus, some individuals may simply have a genetic predisposition to developing wider venules [32].

Key study strengths include the relatively large population-based sample, collection of a substantial amount of confounder information and the quantitative computer-assisted measurement of retinal vessel diameters from digitized fundus photographs. Limitations of this study need to be noted. A major drawback is that our questions on head injury and concussion were relatively crude, for example, we were not able to ascertain mechanism of injury (e.g falls, motor vehicle crash), Glasgow Coma Scale scores, date of occurrence of the head injury and/or concussion, duration of loss of consciousness, and post-traumatic amnesia which precludes us from establishing the type and severity of TBI in this cohort. Hence, although we specifically asked about ‘serious’ head injury or an alteration in consciousness through being knocked out, this cannot be confirmed due to the design of our study. Second, we conducted only cross-sectional analysis and therefore, cannot comment on a ‘cause and effect’ relationship. Although, the most likely direction of association is that TBI preceded changes to the retinal microvasculature, because the reverse direction of effect (retinal venular widening influencing the likelihood of TBI) is unlikely. Third, there are likely to be many potential confounding factors that we may not have adequately adjusted for or did not measure in this study, which could explain the observed associations. For instance, cerebral blood flow changes may be due to other co-morbidities or factors that can also potentially cause an unspecified loss of consciousness, for which participants have not been screened for.

We highlight that the difference in retinal venular caliber between those with a serious head injury/ being knocked unconscious versus the non-injured group was modest (approximately 2 μm). Nevertheless, we and others have shown that even such small differences in retinal vessels can be associated with moderate changes, for example, each 10 mmHg increase in systolic BP was associated with a 1.1 μm reduction in arteriolar caliber [38, 39]. The relationship of retinal vessel caliber is also graded and small differences in adulthood can translate into meaningful differences in disease risk [40].

Therefore, findings from the current exploratory study are encouraging as it suggests that the retinal vascular structure could be a target for further investigations of micro-vessel abnormality in individuals diagnosed with TBI. However, further large prospective studies are needed to examine the association between TBI and long-term changes to the retinal microvasculature. These future studies should also incorporate quantification of retinal network morphology and vessel geometry allowing the assessment of optimality and comparison of networks of healthy individuals and TBI patients. If our findings are confirmed in larger cohorts, there is potential for retinal imaging to be used to identify neurodegenerative processes (fundamentally reflecting neuropathology *in vivo*) early and their effect on progressive anatomical and microstructural changes in the brain after TBI [4].

In summary, our study provides novel epidemiological evidence of an independent association between a serious head injury or being knocked unconscious in older adults and wider retinal venules. However, no significant differences in retinal vessel caliber were observed with those who reported a serious head injury/ knocked unconscious as well as doctor diagnosed concussion. Hence, our findings warrant further research to increase our understanding of the association between microvascular alterations of the retina and TBI. If our findings are validated and confirmed by other large cohort studies, these data could potentially enable the development of novel retinal imaging biomarkers which could assist in the early recognition or better pre-empting of poor outcomes following TBI.

## Supporting information

S1 File Blue Mountains Eye Study Database. Relevant data used to analyze associations between head injury and concussion with retinal vessel caliber.
(XLSX)

S2 File Blue Mountains Eye Study Description. Description of variables used in the analyses.
(DOCX)
