# Supplementary material for: Association between head injury and concussion with retinal vessel caliber
Source: PLoS One. 2018 Jul 11;13(7):e0200441. doi: 10.1371/journal.pone.0200441 (PMC6040728; doi:10.1371/journal.pone.0200441)
Supplement: S2 File — Description of variables used in the analyses. (DOCX) [file pone.0200441.s002.docx]

| **Variables in Creation Order** | | | | |
| --- | --- | --- | --- | --- |
| **#** | **Variable** | **Type** | **Length** | **Label** |
| **1** | IDNUM | Char | 4 | ID Number |
| **2** | SEX | Num | 8 | 1-Women, 2-Men |
| **3** | Age | Num | 8 |  |
| **4** | HEADINJ | Num | 8 |  |
| **5** | CONCUSS | Num | 8 | Concussion, 1 - Yes, 0 - No |
| **6** | WHIPLASH | Num | 8 |  |
| **7** | Diab | Num | 8 | Diabetes, 1 - Yes, 0 - No |
| **8** | SystBP | Num | 8 |  |
| **9** | DiasBP | Num | 8 |  |
| **10** | Smoker | Num | 8 | Smoking,1 - Yes, 0 - No |
| **11** | BMI | Num | 8 |  |
| **12** | MMSE | Num | 8 |  |
| **13** | MAP | Num | 8 |  |
| **14** | HdInConc | Num | s | Severe head injury, |
| **15** | AdjCrae | Num | 8 |  |
| **16** | AdjCrve | Num | 8 |  |
